# Supplementary material for: Regenerative Superhydrophobic Coatings for Enhanced Performance and Durability of High-Voltage Electrical Insulators in Cold Climates
Source: Materials (Basel). 2024 Apr 2;17(7):1622. doi: 10.3390/ma17071622 (PMC11012825; doi:10.3390/ma17071622)
Supplement: Supplementary file 1 [file materials-17-01622-s001.zip › materials-2913217-supplementary Figures.pdf]

materials

MDPI

Article

Regenerative Superhydrophobic Coatings for Enhanced Performance and Durability of High-Voltage Electrical Insulators in Cold Climates

Helya Khademsameni, Reza Jafari *, Anahita Allahdini and Gelareh Momen

Department of Applied Sciences, University of Quebec in Chicoutimi (UQAC), 555 Boul de l'Université, Chicoutimi, QC G7H 2B1, Canada

* Correspondence: rjafari@uqac.ca; Tel.: +1-418-545-5011 (ext. 5635)

Supplementary Materials:

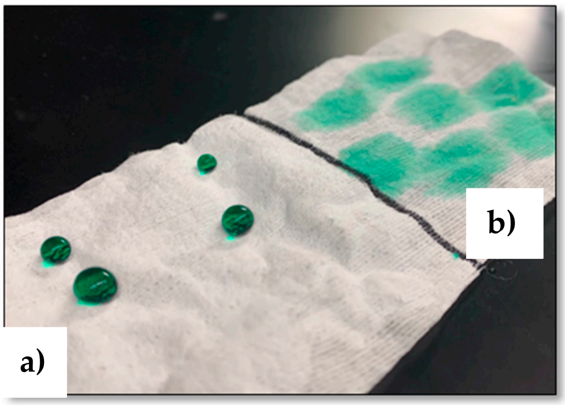

Figure S1. a) Demonstration of the developed SHP–REG coating applied to fabric and b) a pristine fabric lacking the coating.

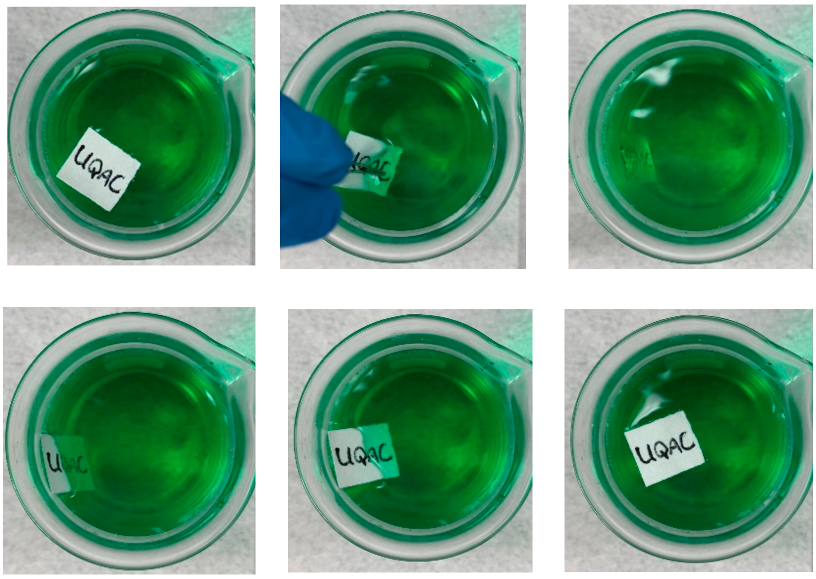

Figure S2. The water-repellent nature of the SHP–REG, which resulted in the coated fabric floating on the surface.

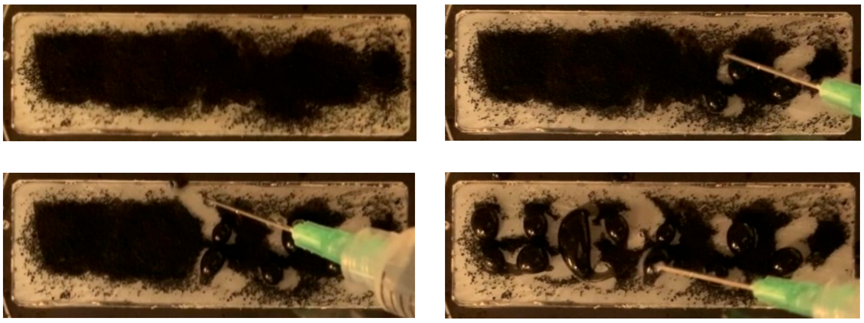

Figure S3. Contamination adhering to the pristine areas and the superhydrophobic areas able to be washed off by the water droplets.

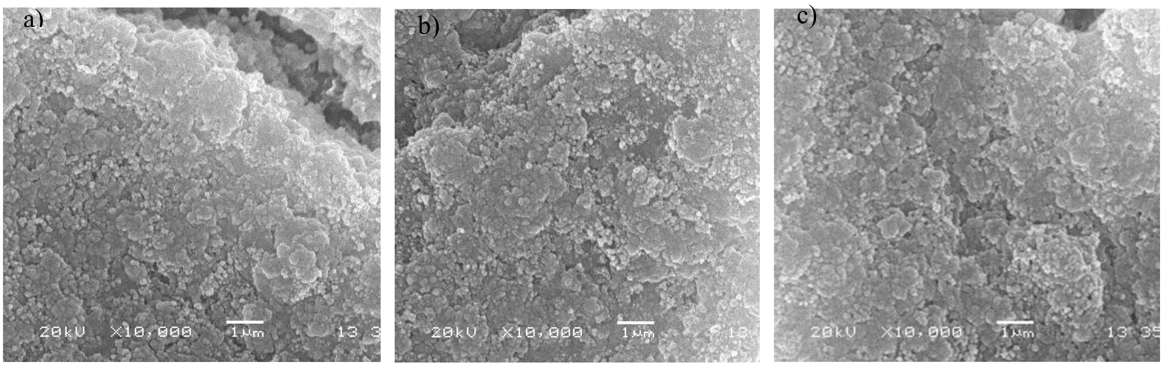

Figure S4. SEM images (×10,000 magnification) of the developed regenerative superhydrophobic coating; a) pristine coating; b) after plasma deterioration with loss of superhydrophobicity; and c) after the regeneration of superhydrophobicity.
